# Macro- and micro-structural insights into primary dystonia: a UK Biobank study

**DOI:** 10.1007/s00415-023-12086-2

**Published:** 2023-11-23

**Authors:** Claire L. MacIver, Grace Bailey, Pedro Luque Laguna, Megan E. Wadon, Ann-Kathrin Schalkamp, Cynthia Sandor, Derek K. Jones, Chantal M. W. Tax, Kathryn J. Peall

**Affiliations:** 1 Division of Psychological Medicine and Clinical Neurosciences, Neuroscience and Mental Health Research Institute, Cardiff University School of Medicine, Cardiff, UK; 2 Cardiff University Brain Imaging Centre (CUBRIC), Cardiff University, Cardiff, UK; 3 Division of Psychological Medicine and Clinical Neurosciences, UK Dementia Research Institute, Cardiff University, Cardiff, UK; 4 Image Sciences Institute, University Medical Center Utrecht, Utrecht, The Netherlands

**Keywords:** Dystonia, MRI, Diffusion MRI, Structural MRI

## Abstract

**Background:**

Dystonia is a hyperkinetic movement disorder with key motor network dysfunction implicated in pathophysiology. The UK Biobank encompasses > 500,000 participants, of whom 42,565 underwent brain MRI scanning. This study applied an optimized pre-processing pipeline, aimed at better accounting for artifact and improving data reliability, to assess for grey and white matter structural MRI changes between individuals diagnosed with primary dystonia and an unaffected control cohort.

**Methods:**

Individuals with dystonia (*n* = 76) were identified from the UK Biobank using published algorithms, alongside an age- and sex-matched unaffected control cohort (*n* = 311). Grey matter morphometric and diffusion measures were assessed, together with white matter diffusion tensor and diffusion kurtosis metrics using tractography and tractometry. Post-hoc Neurite Orientation and Density Distribution Imaging (NODDI) was also undertaken for tracts in which significant differences were observed.

**Results:**

Grey matter tremor-specific striatal differences were observed, with higher radial kurtosis. Tractography identified no white matter differences, however segmental tractometry identified localised differences, particularly in the superior cerebellar peduncles and anterior thalamic radiations, including higher fractional anisotropy and lower orientation distribution index in dystonia, compared to controls. Additional tremor-specific changes included lower neurite density index in the anterior thalamic radiations.

**Conclusions:**

Analysis of imaging data from one of the largest dystonia cohorts to date demonstrates microstructural differences in cerebellar and thalamic white matter connections, with architectural differences such as less orientation dispersion potentially being a component of the morphological structural changes implicated in dystonia. Distinct tremor-related imaging features are also implicated in both grey and white matter.

**Supplementary Information:**

The online version contains supplementary material available at 10.1007/s00415-023-12086-2.

## Introduction

Dystonia is a hyperkinetic movement disorder involving involuntary, repetitive or sustained muscle contractions, estimated to affect up to 120/100,000 population [4]. Dystonia may arise as a primary disorder, or part of a wider neurodevelopmental or neurodegenerative condition, with both genetic and idiopathic forms, and a spectrum of motor distributions. Although the pathophysiological mechanisms of dystonia remain uncertain, animal models implicate disrupted striatal GABA transmission [15] and cerebellar Purkinje cell ectopic dendritic spines [21], while stem cell models have noted functional hyperexcitability, changes to dendritic branching patterns and abnormalities in synaptic plasticity and cellular stress response pathways [29, 32]. Other neurotransmitter systems have additionally been implicated, particularly within the striatal structures, with decreased and irregular cholinergic interneuron firing and reduced inhibitory effect of dopamine receptor agonists [26, 43].

Human neuroimaging has demonstrated reduced network-based functional connectivity between the basal ganglia, sensorimotor and frontoparietal regions [6], alongside disruption to neuronal inhibitory/excitatory balance, with reduced GABA binding and lower post-movement event-related synchronisation of beta activity [14, 33]. Volumetric MRI has indicated higher volumes in task-specific forms of dystonia, most notably in the cerebellar, basal ganglia and sensorimotor grey matter regions [34], with variable findings in non-task-specific forms. Findings from diffusion MRI (dMRI) studies have been mixed, although cerebellar white matter projections, and white matter deep to the primary sensorimotor cortices have been highlighted [23]. There is also evidence for genotypic and phenotypic variation, including more widespread changes in genetic dystonias and intermediate changes in non-motor manifesting gene carriers. In addition, most existing literature uses non-specific analysis strategies such as fractional anisotropy (FA) [23] averaged along a neuronal tract, with these providing limited biological insight into the pathological underpinnings.

**Fig. 1:**
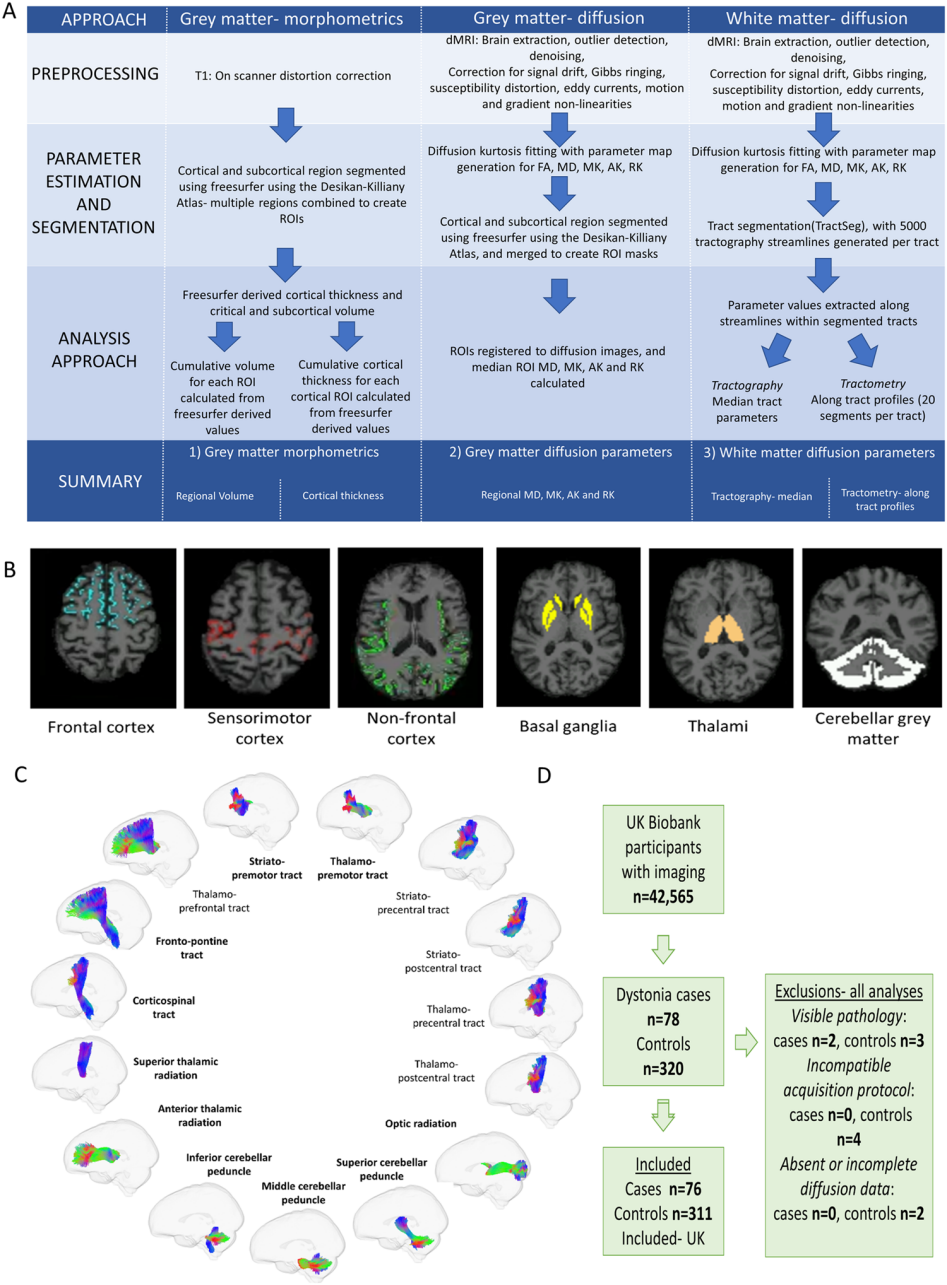
Schematic of methodology. **a** outline of analysis methodology; **b** grey matter regions of interest; **c** white matter tracts assessed with tractography; those shown in bold are used in the tractometry analysis; **d** recruitment flowchart showing final inclusions and exclusions. *dMRI* diffusion magnetic resonance imaging; *DKI* diffusion kurtosis imaging; *ROI* region of interest; *FA* fractional anisotropy; *MD* mean diffusivity; *MK* mean kurtosis; *AK* axial kurtosis; *RK* radial kurtosis

The UK Biobank (http://www.ukbiobank.ac.uk) is a prospective database containing health-related data for > 500,000 participants across the UK, with previous work identifying 1572 individuals diagnosed with dystonia within this cohort [38]. Brain MRI was undertaken in a subset of the overall cohort (*n* = 42,565) including T1-weighted and multi-shell diffusion weighted sequences [8]. Here, we aim to assess whether diffusion based microstructural MRI parameters differ in key motor grey matter, notably cerebellar, basal ganglia, thalamic and sensorimotor grey matter, and their interconnecting white matters pathways, in the dystonia cohort in the UK Biobank compared to age- and sex-matched unaffected controls. Additionally, and in light of the suggestion from previous studies that imaging changes may differ across distinct dystonia subtypes [7], we have also analyzed the largest individual diagnostic groups—cervical dystonia and dystonic tremor—again, with comparison to the control cohort (Fig. 1).

## Methods

### Cohort derivation and characteristics

Identification of the UK Biobank dystonia cohort used a clinically validated algorithm derived from a national clinically linked databank [4, 38]. This made use of ICD-10 codes derived from NHS hospital records (UK Biobank data fields 41,202, 41,204 and 41,207) and Primary care records (Read codes v2, data portal field read_2), with inclusion and exclusion diagnoses outlined in Supplementary Table 1. All individuals identified as having been diagnosed with dystonia and who had undergone T_1_-weighted and diffusion-weighted brain MRI were included (UK Biobank application number 69610). A 1:4 age- and sex-matched control cohort was derived from the overall cohort following application of the same exclusion criteria as was used for identification of the dystonia cohort [38]. Scans marked by the UKBB as ‘unusable’ (based on pathology or T1 artifact) were visually inspected and only excluded if pathology was identifiable. Participants were excluded from the diffusion-based analyses if they had missing or incomplete diffusion-weighted imaging data or were marked by the UKBB as ‘incompatible’ due to use of a different acquisition protocol.

### Imaging acquisition

Imaging was undertaken in one of four centres (Table 1) using a 3 T Siemens Skyra scanner with a standard Siemens 32-channel radiofrequency receive head coil, using a standardised acquisition protocol across sites. A sagittal 3D T_1_-weighted MPRAGE was acquired, with field of view 208 × 256 × 256 and voxel size 1mmx1mmx1mm. The dMRI acquisition used a spin echo planar imaging sequence with × 3 multi-slice acceleration, fat saturation, field of view 104 × 104 × 72, and voxel dimensions 2mm × 2mm × 2mm. Fifty distinct diffusion directions were acquired each for b = 1000 s/mm^2^ and b = 2000s/mm^2^, with 5 b = 0 s/mm^2^ images. A 6/8 partial Fourier readout was used [1, 24].

**Table 1:** Participant demographics and dystonia subgroups

Group	Number of cases	Male: Female (*n*)	Median Age- years (s.d.)	Number of individuals scanned at individual MRI locations (Cheadle/Reading/Newcastle/Bristol)
Unaffected control cohort	311	132:179	64.62 (8.93)	40/22/14/0
Whole dystonia cohort	76	31:45	64.16 (8.75)	180/44/86/1
Idiopathic torsion dystonia	0	–	–	–
Idiopathic non-familial dystonia	0	–	–	–
Idiopathic familial dystonia	0	–	–	–
Cervical dystonia	40	17:23	62.68 (8.87)	23/9/8/0
Idiopathic orofacial dystonia	2	2:0	58 (11.31)	0/2/0/0
Blepharospasm	9	3:6	72 (7.98)	4/2/1/0
Writer’s cramp	0	–	–	–
Myoclonic dystonia	0	–	–	–
Segawa syndrome	0	–	–	–
Unspecified tremor	17	10:7	68.53 (8.33)	7/7/3/0
Unspecified dystonia	10	0:10	61 (6.52)	6/2/2/0

### dMRI preprocessing and parameter estimation

dMRI data are processed within the UK Biobank using standardised pipelines. However, significant variability of approach exists in diffusion imaging analysis, and while there is no current evidence based consensus on optimal approaches, attempts have been made to optimize methodology to reduce the impact of imaging artefacts on dMRI data [2]. For this study, a local, optimized preprocessing pipeline was applied to the raw diffusion data (with facial feature removal prior to UK Biobank data release), described in Fig. 1A. This involved brain extraction, denoising [35], outlier detection (e.g., due to signal dropout from motion) [31], signal drift correction, and correction for Gibbs ringing [18], eddy currents, susceptibility distortion and subject motion [3], and gradient non linearities [5]. Each of the analytical steps were manually reviewed for a randomly selected 10% subset to assess data quality. Parameter estimation used a diffusion kurtosis representation and a constrained weighted least squares fitting approach with weight reduction for outlier measurement [30, 37], demonstrated to enable more accurate estimation of diffusion tensor imaging (DTI) parameters [36] than more standard DTI fitting approaches. Parameter maps were created for FA, mean diffusivity (MD), mean kurtosis (MK), axial kurtosis (AK) and radial kurtosis (RK). These parameter maps were additionally used in the grey matter analysis detailed below.

### Analysis approach

### Grey matter analysis

The raw T_1_-weighted images were used for grey matter segmentation using Freesurfer [13], with six region of interest (ROI) masks created from these segmentations (prefrontal, sensorimotor, non-frontal, striatal, thalamic and cerebellar cortical masks, Fig. 1A & B, Supplementary Table 2). Within each ROI, total cortical thickness (summed subregion values) and total volume were calculated, with the latter also calculated for subcortical regions. Grey matter diffusion measures were calculated with the first b = 0 image registered to the brain extracted T_1_-weighted image for each participant (using FSL EPI reg [17]), and the inverse of this transform applied to the GM ROIs with nearest neighbour interpolation into diffusion space. Voxels with partial volume with other tissues due to the differing voxel dimensions were excluded and median ROI MD, MK, AK and RK were calculated (from the diffusion kurtosis parameter maps).

### White matter analysis

Within the white matter pathways, we evaluated both whole tract averages (tractography) and along-tract profiling (tractometry), investigating evidence of localised changes in tract morphology, as has been shown in genetically homogenous dystonia cohorts [9]. Tractseg [40] was used to segment the motor tracts: middle cerebellar peduncle, bilateral inferior cerebellar peduncles, superior cerebellar peduncles, frontopontine tracts, corticospinal tracts, anterior thalamic radiations, superior thalamic radiations, thalamoprefrontal tracts, thalamopremotor tracts, thalamoprecentral tracts, thalamopostcentral tracts, striatopremotor, striatoprecentral tracts and striatopostcentral tracts (Fig. 1C). Optic radiations were included as a non-motor comparison tract [23]. Multi-shell multi-tissue constrained spherical deconvolution was applied to the preprocessed diffusion images to extract the white matter fODF, with subsequent peak-extraction limited to a maximum of three peaks/voxel. A fully-connected convolutional neural network was used to create a tract probability image for each orientation and tract, with start and end regions segmented and fibre orientation maps calculated within the segmented regions [42]. Probabilistic tractography [41] with 5000 streamlines was performed within each segmented region, with median parameter diffusion tensor values (FA and MD) and diffusion kurtosis values (MK, AK and RK) extracted from the streamlines. In addition, along-tract profiling (tractometry) was performed [11, 39]: the tracts were portioned into 20 segments along their length with the centroid of all streamlines within each segment identified, the end two segments excluded and median value across streamlines calculated for each of the 18 remaining segments [11]. The thalamoprefrontal, thalamoprecentral, striatoprecentral, thalamopostcentral and striatopostcentral tracts were excluded from tractometry analysis due to their geometry not enabling consistent segmentations to be produced.

### Neurite orientation and dispersion density index (NODDI)

Tracts where significant differences were observed between the dystonia and control cohorts were further examined using white matter compartmental parameters (OD—orientation dispersion index, ND—neurite density index, and FWF—free water signal fraction), estimated using post-hoc Neurite Orientation, Dispersion and Density Imaging (NODDI) [44], hypothesized to better disentangle biological features. This was performed for tracts that demonstrated significant differences in any of the original analyses. As the tractometry analyses demonstrated visually corresponding tract profiles for left and right tracts, the two were combined for this post hoc analysis.

### Statistical analysis

Analysis was undertaken using RStudio v0.99.892. Participant demographic information was summarised using descriptive statistics. Simple linear regression models were applied comparing disease status (independent variable) for each parameter and tract (dependent variables). Subgroup analyses were performed for the two largest diagnostic subgroups—cervical dystonia and dystonic tremor—compared to unaffected controls. Bonferroni correction for multiple comparisons was applied for both tracts and parameters, with differences reported only if p-values were significant (*p* < 0.05) following correction.

## Results

### Demographics

Initially, 78 individuals diagnosed with dystonia and 320 control participants with imaging data were identified. Of these, two dystonia and 9 control cases were excluded due to visible pathology (cases *n* = 2 (extensive white matter changes *n* = 1, hydrocephalus *n* = 1), controls *n* = 3 (extensive white matter changes *n* = 2, congenital malformation *n* = 1)), incompatible acquisition protocols (*n* = 4) or absent/incomplete diffusion data (*n* = 2), Therefore, a total of 76 individuals with dystonia and 311 healthy controls were included for onward analysis. Median age at scanning for both patient and control groups was 64 (SD 8.87 and 8.91 respectively), with a male to female ratio of 1:1.44 for both groups. Dystonia cases were subdivided based on available clinical diagnoses, with the two largest groups being cervical dystonia (*n* = 40) and dystonic tremor (*n* = 17) (Table 1).

### Grey matter analysis

**Fig. 2:**
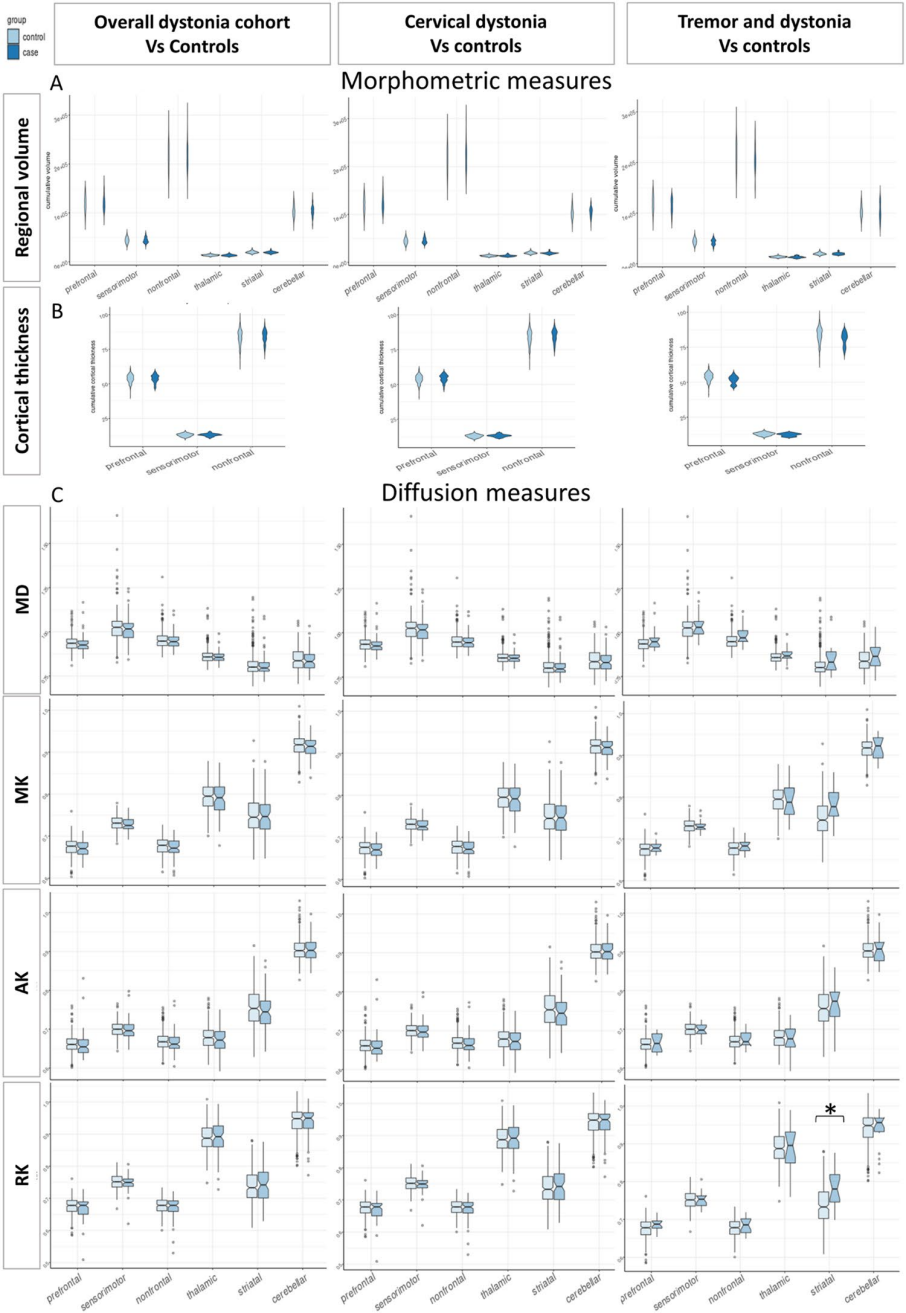
Box plots showing median values for dystonia and controls. **a** regional volume; **b** cortical thickness; **c** Diffusion measures. Significant differences on linear model following multiple comparison correction are highlighted with *

No significant differences in regional volume or cortical thickness were observed when comparing the overall dystonia, cervical or tremor cohorts to unaffected controls. Diffusion kurtosis measures showed significant differences only in the striatum for the tremor cohort, with a higher RK values (*p* = 5.84 × 10^–4^) compared to controls (Fig. 2).

## White matter analysis

### Tractography

No significant diffusion tensor (FA or MD) or diffusion kurtosis (MK, AK, RK) differences were observed between the overall cohort, cervical or dystonic tremor cohorts, compared to the unaffected control group (Supplementary Fig. 1).

### Tractometry

**Fig. 3:**
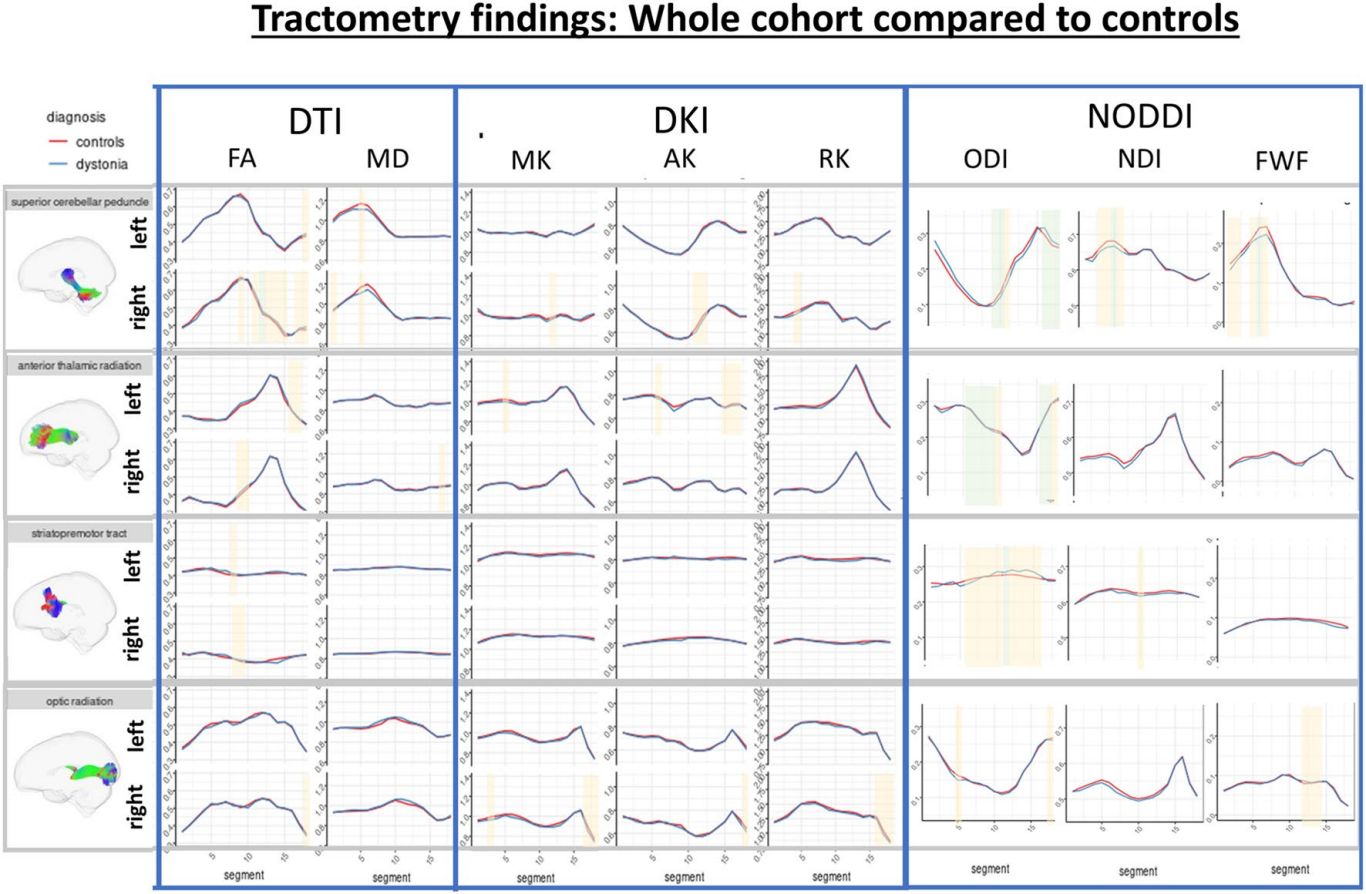
Along tract profiles (tractometry) for the whole dystonia cohort compared to controls. Green shaded regions represent statistically significant linear model results following multiple comparison correction. Yellow shaded regions are significant (*p* < 0.050) prior to correction

**Fig. 4:**
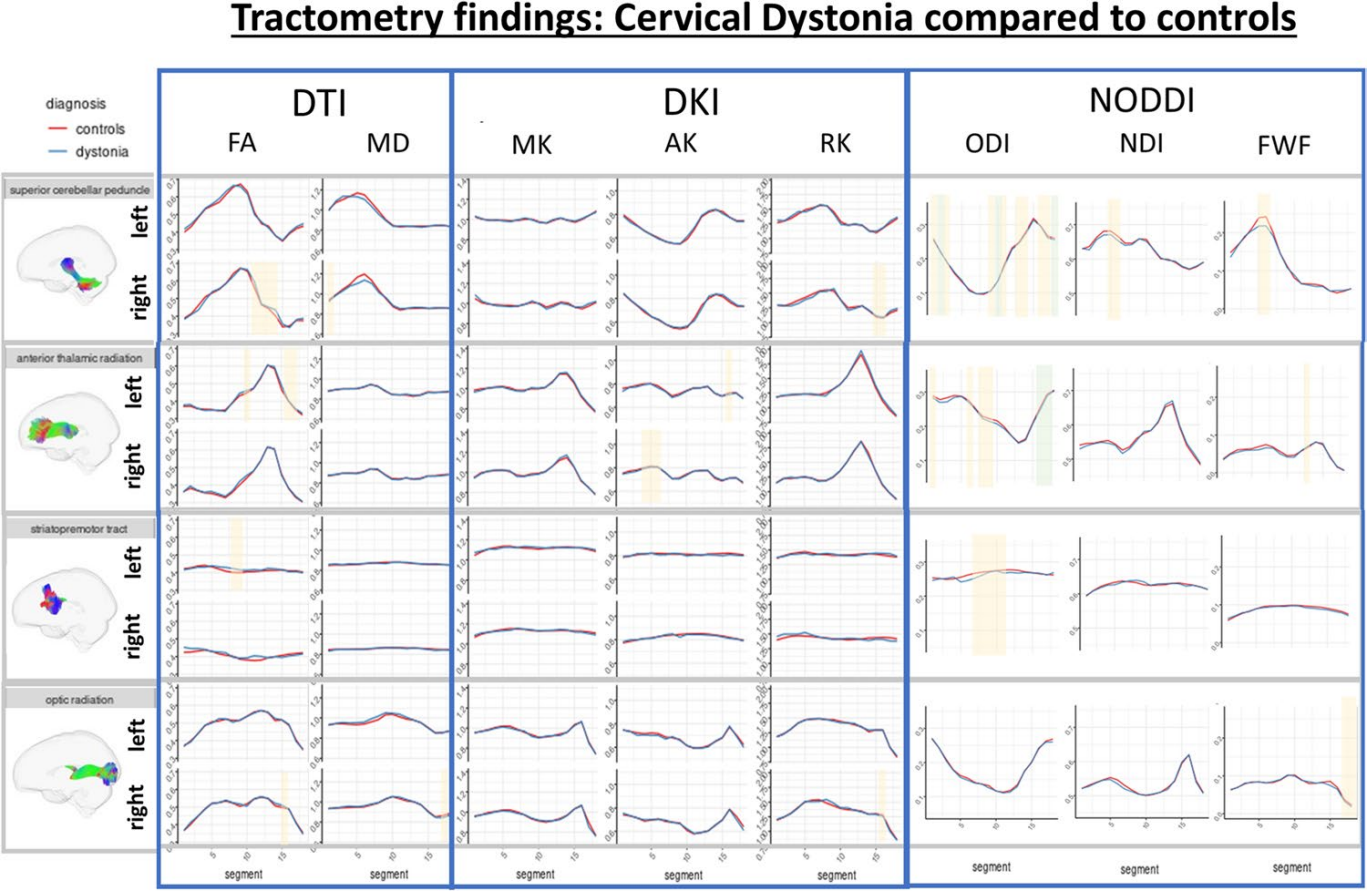
Along tract profiles (tractometry) for the cervical dystonia cohort compared to controls. Green shaded regions represent statistically significant linear model results following multiple comparison correction. Yellow shaded regions are significant (*p* < 0.050) prior to correction

**Fig. 5:**
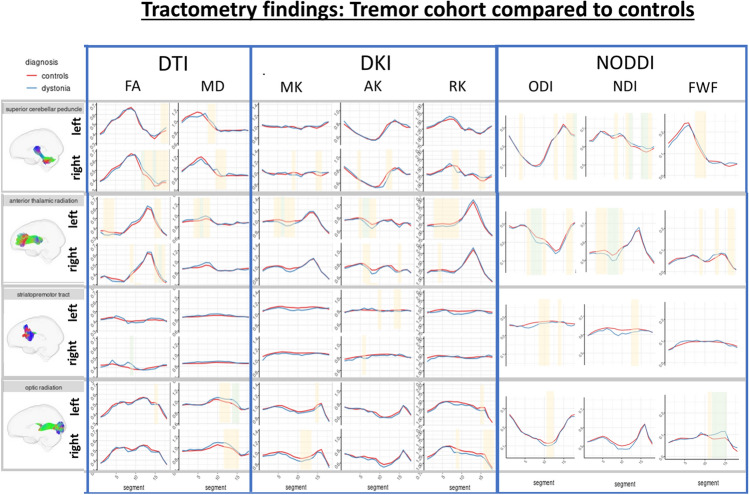
Along tract profiles (tractometry) for the tremor cohort compared to controls. Green shaded regions represent statistically significant linear model results following multiple comparison correction. Yellow shaded regions are significant (*p* < 0.050) prior to correction

For tracts in which significant differences were observed, along-tract profiles are included in Figs. 3, 4, 5 (Supplementary Figs. 2, 3, 4 display all tract analyses).

### Overall dystonia cohort vs. unaffected control group

Figure 3 demonstrates higher FA in the mid right superior cerebellar peduncle (*p* = 2.69 × 10^–4^), while NODDI identified significant changes to ODI measures across the same tract (lower mid-tract (*p* = 7.19 × 10^–4^) and higher distal tract (*p* = 3.3 × 10^–7^), as well as significantly lower NDI (*p* = 6.8 × 10^–4^) and FWF (*p* = 0.002) values in the dystonia cohort. ODI differences were also demonstrated in the anterior thalamic radiations (higher proximal-mid (*p* = 6.53 × 10^–5^) and distal (*p* = 1.96 × 10^–4^) values) and striatopremotor (lower mid-tract values (*p *= 0.001) tracts in the dystonia cohort.

### Cervical dystonia cohort vs. unaffected control group

No significant differences were observed for DTI and DKI measures (Fig. 4). NODDI identified differences in ODI in the superior cerebellar peduncles (higher proximal (*p* = 0.002) and lower mid (*p* = 4.17 × 10^–4^) and distal (*p* = 1.2 × 10^–4^) values), and anterior thalamic radiations (lower distal values (*p* = 7.37 × 10^–4^)) in the cervical dystonia cohort compared to controls Fig. 5.

### Dystonia with tremor vs. unaffected control group

Multiple regions of significant difference were observed across the superior cerebellar peduncle and anterior thalamic radiation, including FA (right, higher in tremor (*p* = 5.22 × 10^–4^)), MD (left, higher in tremor (*p* = 3.43 × 10^–4^)), MK (left, lower in tremor (*p* = 4.59 × 10^–4^)) and AK (left, lower in tremor (*p* = 1.92 × 10^–4^)) in the anterior thalamic radiations, with higher FA (*p* = 6.61 × 10^–5^) found in the right superior cerebellar peduncle. These were coupled with corresponding changes in NODDI measures, with lower ODI (*p* = 4.31 × 10^–4^) and higher NDI (*p* = 8.44 × 10^–5^) in the superior cerebellar peduncles, and lower ODI (*p* = 8.3 × 10^–5^) and lower NDI (*p* = 5.96 × 10^–4^) in the anterior thalamic radiations. Differences were additionally observed in the right striatopremotor tract (right tract, higher FA (*p* = 4.71 × 10^–4)^)) and optic radiations (left tract, higher FA (*p* = 1.78 × 10^–7^), and higher distal FWF (*p* = 1.6 × 10^–7^)) in the dystonic tremor group compared to controls.

## Discussion

**Fig. 6:**
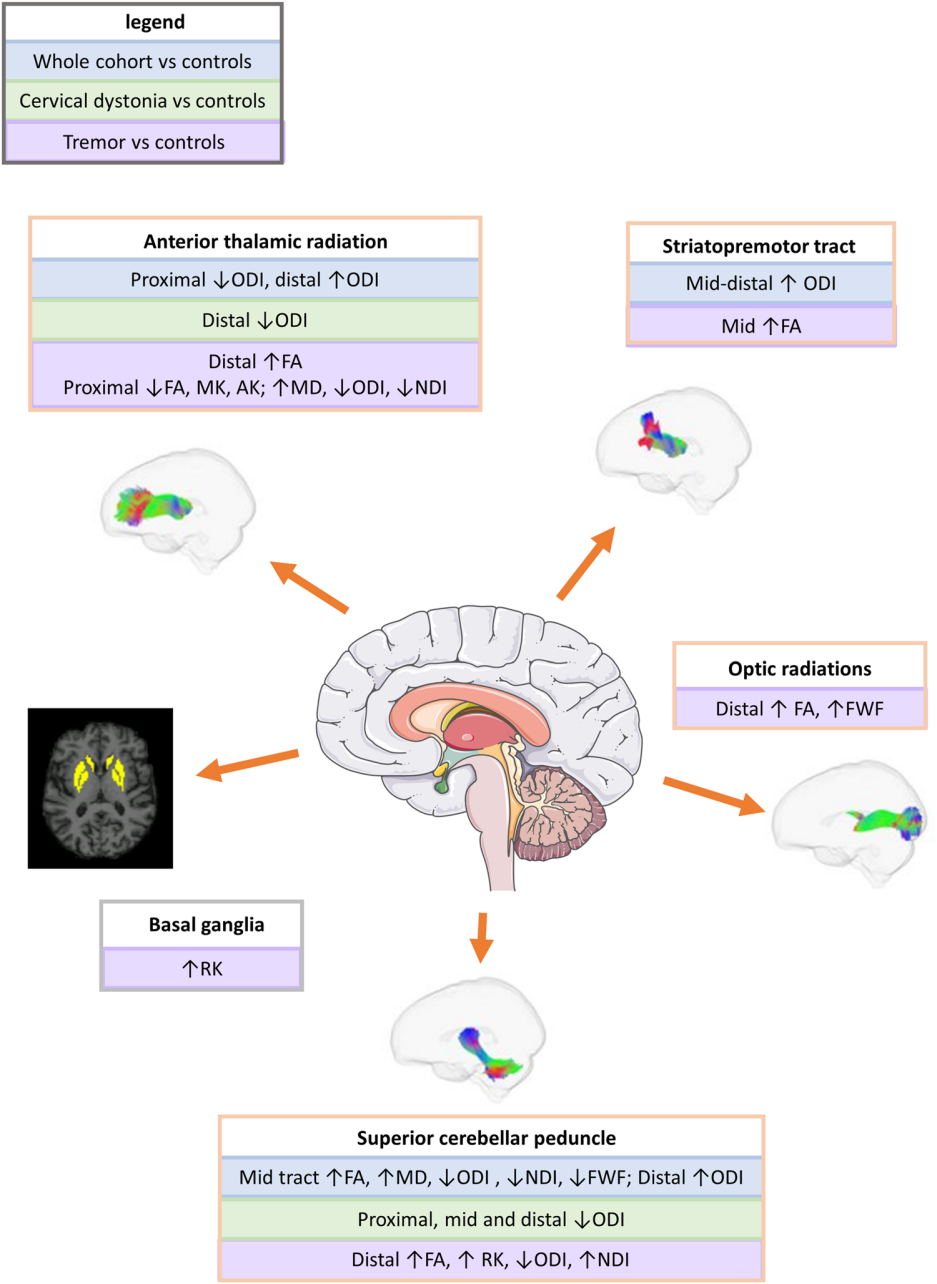
Summary of key findings. *FA* fractional anisotropy; *MD* mean diffusivity; *MK* mean kurtosis; *AK* axial kurtosis; *RK* radial kurtosis; *ODI* orientation distribution index; *NDI* neurite density index

This study analyses T1- and diffusion-weighted brain MRI in a large dystonia cohort, identified from the UK Biobank cohort, using an optimized imaging processing approach. It provides evidence of both grey and white matter microstructural differences in key motor networks in dystonia, with striatal grey matter kurtosis differences, and localised white matter differences in key motor tracts, notably the superior cerebellar peduncle, anterior thalamic radiations and striatopremotor tracts (Fig. 6).

### Grey matter 

Morphometric analyses demonstrated no between group differences for the overall or subgroup analyses. This is partially at odds with existing literature, particularly in genetic and task specific forms of dystonia [16, 27]. However, non-task-specific dystonias, such as cervical dystonia and blepharospasm, have demonstrated substantial variability in both the presence and direction of any identified volumetric differences. Minimal existing work has investigated GM in dystonic tremor, identifying increases in the inferior frontal cortex [19], motor cortex [10] and striatal [19] volumes/thickness. Not previously applied in a dystonia cohort, grey matter diffusion kurtosis measures here identified tremor-specific striatal differences, with significantly higher RK values. Previous work has shown diffusion kurtosis measures to be correlated with brain microstructural changes, including during normal brain development [25] and in white matter in multiple sclerosis [22]. The differences observed may indicate subtle morphological striatal changes, particular to tremor, such as in cell body density or dendritic branching complexity, however, the MR acquisition methodology used as part of the UK Biobank study is insufficient to apply modelling approaches to ascertain the nature of this signal difference. Approaches which provide additional information on tissue heterogeneity, including compartmentalisation into neurites and soma, may facilitate better understanding and insight into these observed differences.

### White matter

Tractography identified no significant differences between groups, although multiple tracts had significantly higher FA values in the dystonia cohort compared to controls prior to correction for multiple comparisons. Tractometry, aimed at identifying regional variation along individual tracts, identified localized differences, most notably higher FA in the superior cerebellar peduncles, anterior thalamic radiations and striatopremotor tracts (Fig. 6). These higher FA values are potentially explained by disrupted inhibition resulting in increased pathway activation and subsequent increased muscle activity, or conversely could reflect a mechanism of attempted compensation for muscle overactivation. While studies involving cohorts of individuals diagnosed with genetic dystonias have often identified lower (rather than higher) FA values, those involving idiopathic forms (comprising the majority of this cohort) have reported more mixed results with some identifying lower MD or higher FA in corroboration with our findings, while only a few have found lower FA values [7, 12, 28]. The dissonance may reflect the heterogeneity of different dystonia diagnoses with varying underlying pathological processes, including, for example, disruption to white matter tract integrity in some forms, with others such as adult onset idiopathic dystonias involving differing mechanism such as reinforcement of selected projections. Alternatively, aberrance of earlier life neurodevelopmental processes may be involved, such as the pruning of dendritic connections and increases in the complexity of WM pathway course, which later decompensate and clinically manifest as a focal dystonia. The optic radiations, included as a non-motor comparison, unexpectedly demonstrated distally higher FA in the tremor cohort, with a few studies previously implicating visual regions in dystonia [28]. This may reflect compensatory visual system processes given the role of visual feedback in movement, or evidence of more diffuse microstructural changes within the brain.

Post hoc NODDI was undertaken as it is hypothesized to provide more microstructurally meaningful interpretation of signal differences, demonstrating more pronounced localised differences in the tracts implicated in DTI and DKI analysis. The superior cerebellar peduncles and thalamic radiations were the most dominantly implicated, with ODI the measure in which greatest variation between groups was observed, with overall lower values in the dystonia cohort. This corresponds with the FA differences outlined above, and the suggestion of ‘over-use’ of subsets of neuronal pathways in giving rise to dystonia. Further interpretation of the ODI NODDI measure could suggest that white matter changes in idiopathic dystonia are related to disruption to the organisation and dispersion of fibres within tracts, rather than differences in axonal morphology. However,—in the superior cerebellar peduncles for the overall dystonia cohort, and for the superior cerebellar peduncles, anterior thalamic radiations and the optic radiations for the tremor cohort compared to controls—differences in NDI and FWF measures were also observed, potentially indicating a degree of morphological change to the axons themselves in these regions, which may reflect secondary adaptation or an alternative primary pathological process. No previous work has applied NODDI in a dystonia cohort for comparison, although one fixel-based analysis approach has identified a lower Fibre Density value in the striatal region [45].

Differences, compared to the control cohort, were most frequently observed in the tremor cohort, observed across both grey and white matter measures. Several more recent studies have indicated that distinct imaging changes are observed when both tremor and focal dystonia are observed, compared to isolated, focal dystonia alone. Suggested explanations for this include adaptive changes, particular to tremor, rather than of the dystonic syndrome itself. The limited dystonia subgroup sizes and phenotypic detail available within the UK Biobank dataset doesn’t allow for a direct tremor versus no tremor comparison, however, a previous comparison of dystonic and essential tremor identified higher FA in cerebellar projections, regardless of tremor type but to a greater extent within the dystonic tremor cohort, with wider evidence existing of differences between tremulous and non-tremulous dystonia forms [7]. One limitation of the limited phenotypic data available in the UK Biobank is the inability to determine body part affected by tremor, with this broad diagnostic category likely encompassing multiple phenotypic subgroups which may have differing imaging correlates. Additionally, whilst a diagnosis of essential tremor is applied as an exclusion criteria (Supplementary material 1), there is potential that a portion of this group would fall into this diagnostic category, and so the tremor group and resultant findings may represent a broader range of the tremor spectrum than dystonic tremor alone.

As we observed, whole tract average tractography approaches potentially overlook localized pathway changes, with along-tract profiling (tractometry) providing more anatomically specific localised assessment, undertaken here for the first time in dystonia. Despite this advantage, diffusion tensor and kurtosis measures remain non-specific even when profiled along a tract. Biophysical models such as NODDI aim to overcome some limitations of traditional DTI and DKI approaches by modelling tissue microstructural properties such as intra- and extra-axonal compartments. However, estimating microstructural parameters with acquisitions at low-to-moderate b-values (as used in this acquisition) is notoriously difficult due to inherent degeneracy (where multiple physically plausible parameter settings result in the same signal decay) and requires a priori fixing or linking of values, limiting the ability to capture true biophysical changes [20]. Hence, these measures should be interpreted with caution, and more extensive acquisitions are necessary to map more specific microstructural features. A further limitation of this dataset is the lack of in-depth clinical phenotyping data available, with the heterogeneous spectrum of dystonic disorders reduced to broad diagnostic categories providing minimal insight into the specific motor (or non-motor) phenotype of the participants. This limits potential interpretation of the findings seen here and requires further work in well phenotyped and homogenous cohorts in future to delineate syndrome and symptom specific differences. This is a particularly dominant factor within the overall cohort analysis, where potentially differences between the dystonia syndromes may have differing impacts, either cancelling or diluting the resultant imaging differences seen. To assess whether the cervical dystonia cases, comprising the majority of the overall cohort, were having excess impact on the overall analysis, we performed a Cohen’s D effect size calculation between the cervical and non-cervical dystonia cases for two key regions, finding no meaningful between group differences (ODI measure in the mid anterior thalamic radiation: 0.029; FA in the mid-distal right superior cerebellar peduncle: 0.097). Additionally, whether the differences seen are causative or compensatory cannot be established via a cross-sectional imaging study, with longitudinal and animal model studies necessary to attempt to disentangle this point. Cross site image acquisition differences have additional potential to influence findings in a study such as this, however, previous work in UK Biobank data has demonstrated that this variation in image centre has had minimal impact on study findings [2].

Overall, this work demonstrates microstructural motor pathway differences in idiopathic forms of dystonia, with additional changes that appear to be specific to when dystonia and tremor are observed together. Further work is needed to develop and apply more biologically meaningful and specific in vivo human imaging measures, in robustly phenotyped clinical cohorts, that may better allow disentanglement of potential underlying brain morphological and microstructural differences and advance mechanistic understanding in dystonia.

### Supplementary Information

Below is the link to the electronic supplementary material.

Supplementary file1 (TIF 2645 KB)

Supplementary file2 (TIF 2779 KB)

Supplementary file3 (TIF 3095 KB)

Supplementary file4 (TIF 2920 KB)

Supplementary file5 (DOCX 24 KB)

Supplementary file6 (DOCX 15 KB)

## Data Availability

The data used for this study is accessable via the UK biobank platform https://ams.ukbiobank.ac.uk/ams/.
