# Supplementary figures and images for: Macro- and micro-structural insights into primary dystonia: a UK Biobank study
Source: J Neurol. 2023 Nov 23;271(3):1416–27. doi: 10.1007/s00415-023-12086-2 (PMC10896800; doi:10.1007/s00415-023-12086-2)

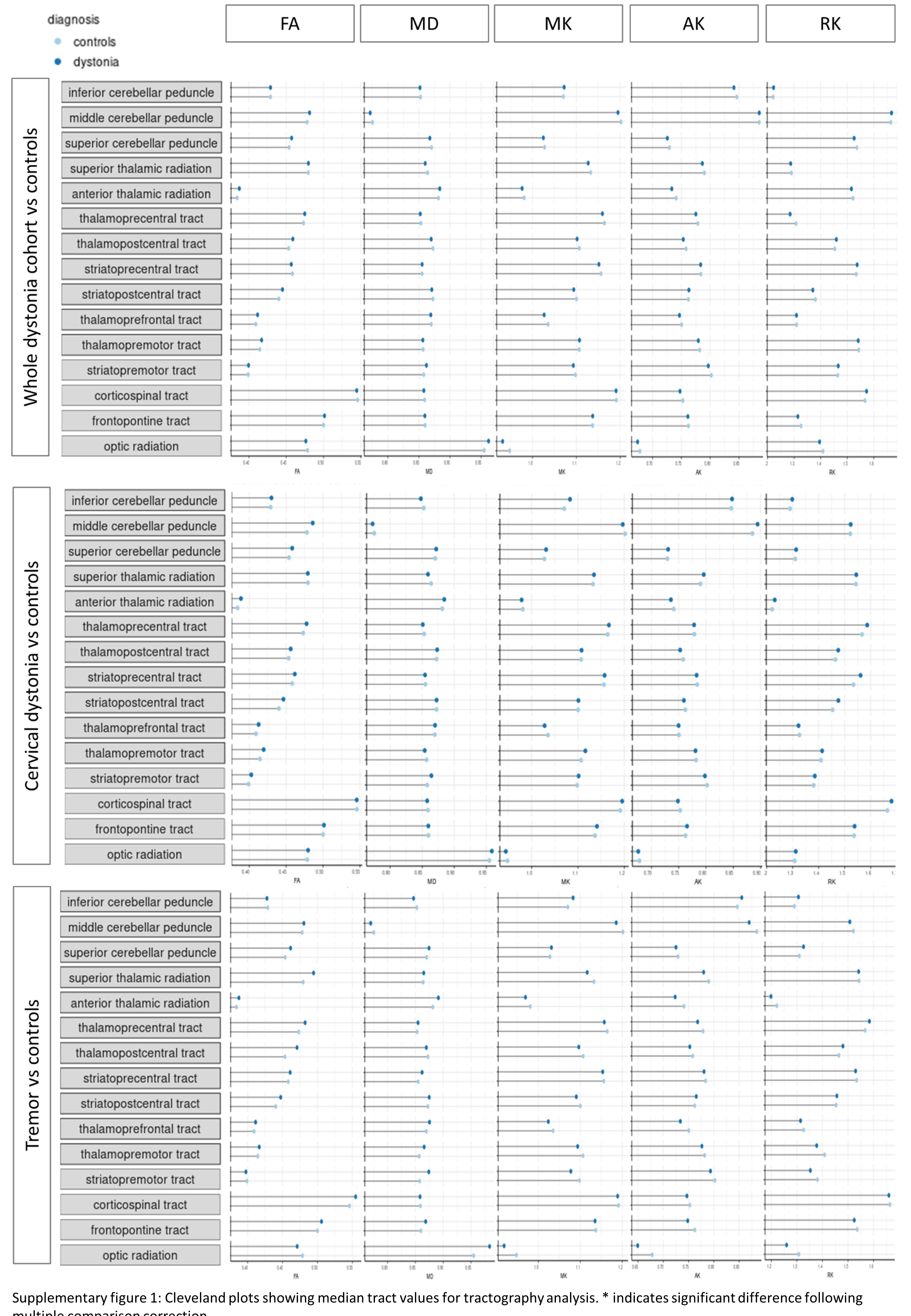

Supplement: Supplementary file 1 — Supplementary file1 (TIF 2645 KB) [file 415_2023_12086_MOESM1_ESM.tif]

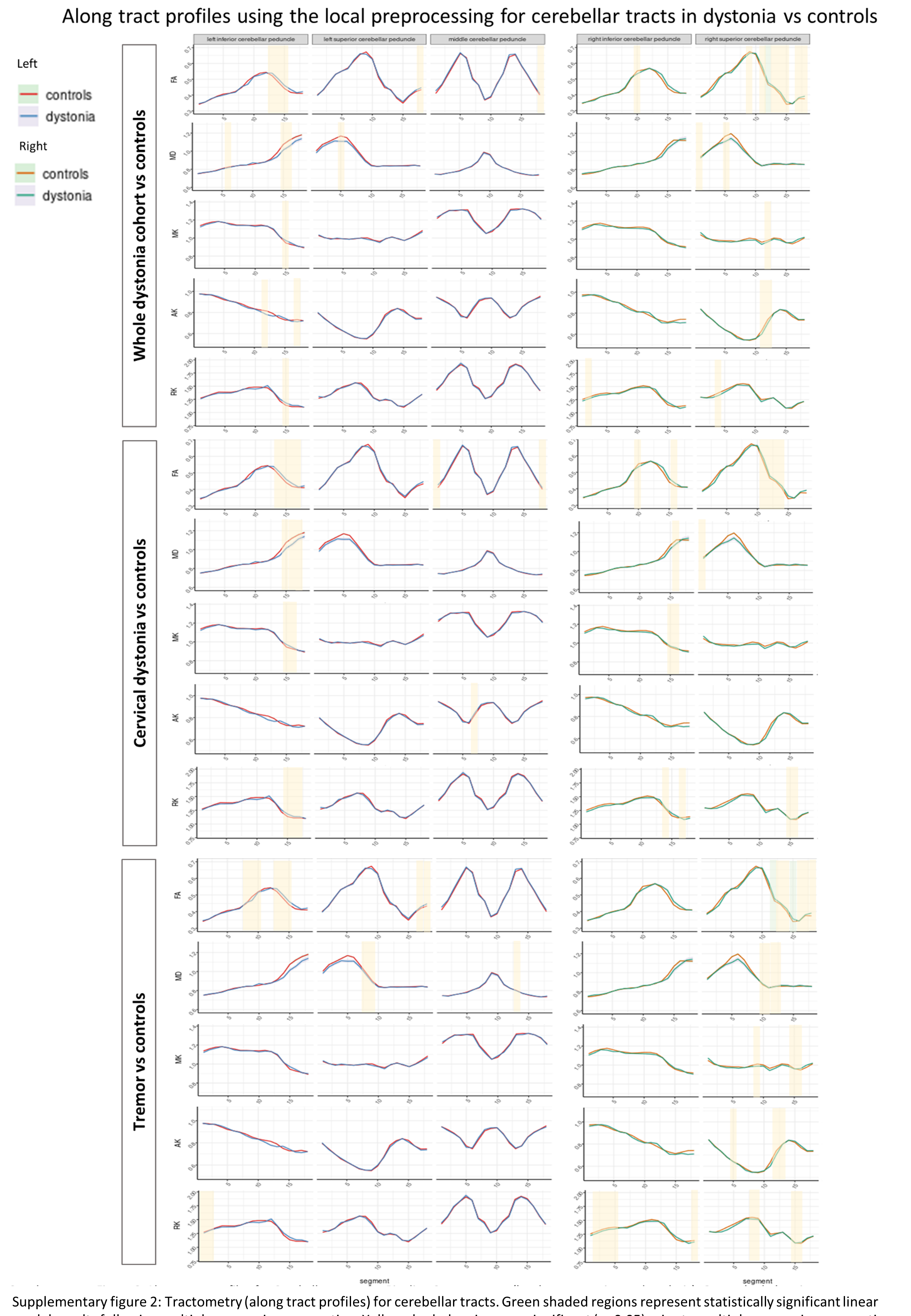

Supplement: Supplementary file 2 — Supplementary file2 (TIF 2779 KB) [file 415_2023_12086_MOESM2_ESM.tif]

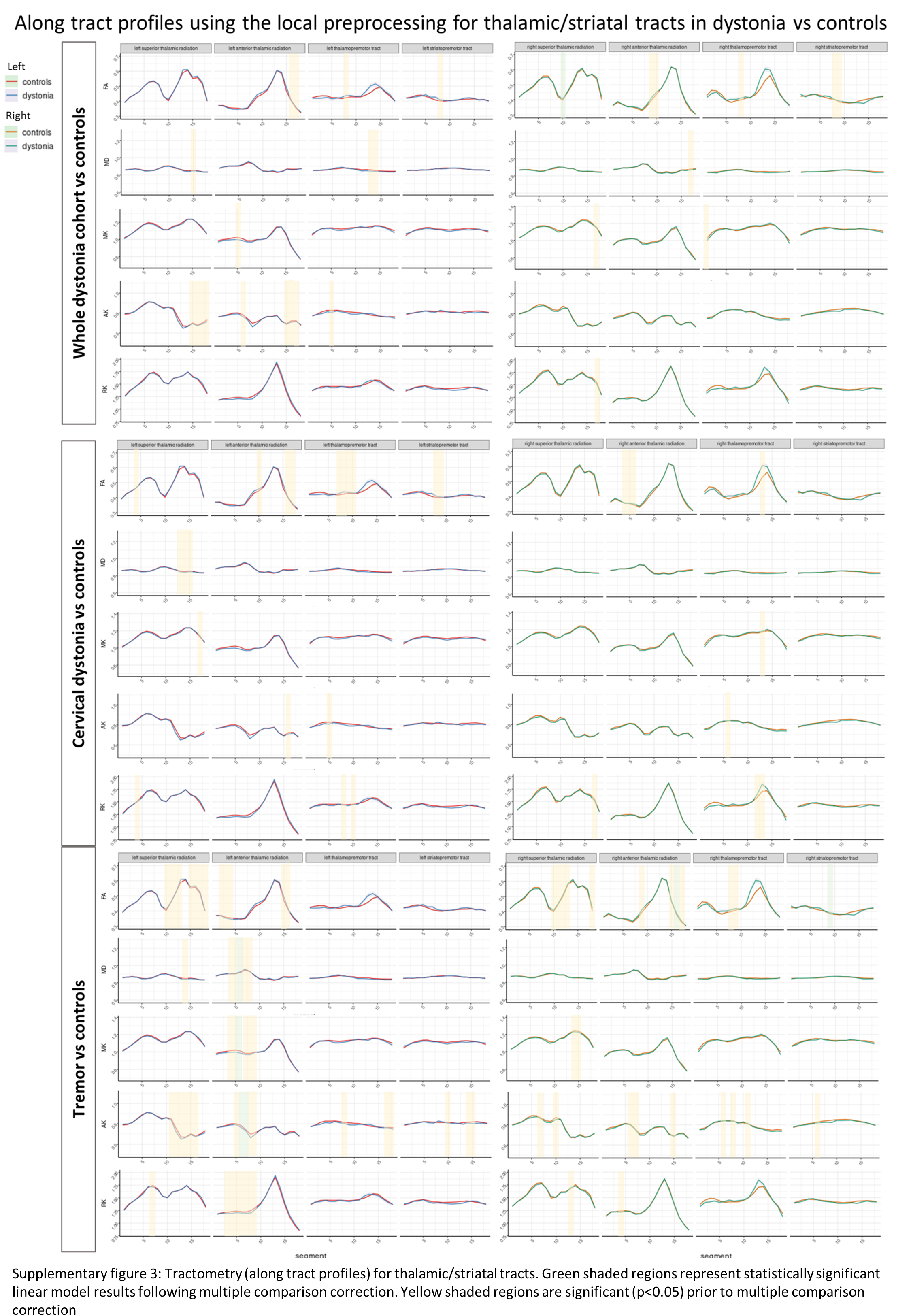

Supplement: Supplementary file 3 — Supplementary file3 (TIF 3095 KB) [file 415_2023_12086_MOESM3_ESM.tif]

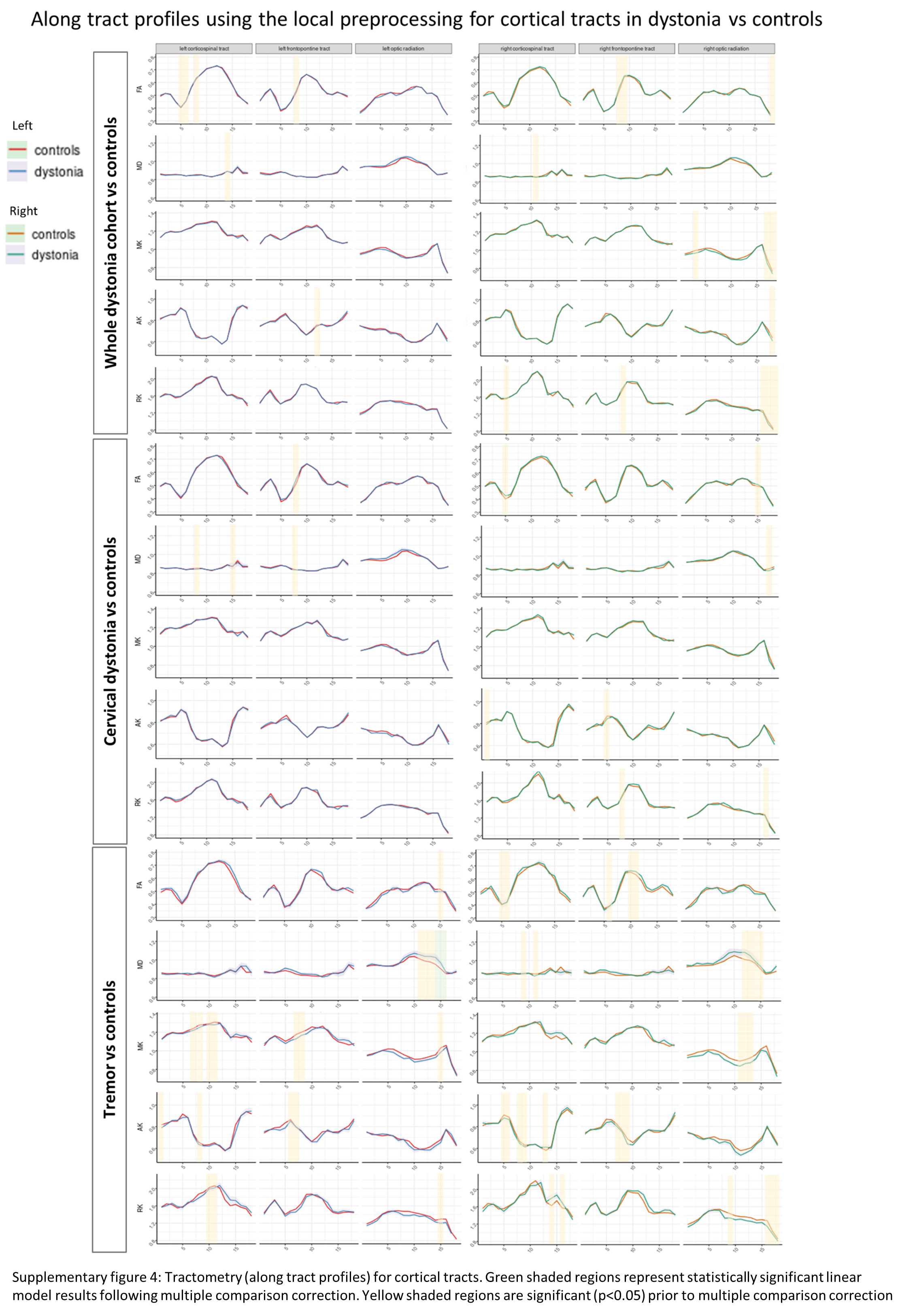

Supplement: Supplementary file 4 — Supplementary file4 (TIF 2920 KB) [file 415_2023_12086_MOESM4_ESM.tif]
