# Supplementary material for: Macro- and micro-structural insights into primary dystonia: a UK Biobank study
Source: J Neurol. 2023 Nov 23;271(3):1416–27. doi: 10.1007/s00415-023-12086-2 (PMC10896800; doi:10.1007/s00415-023-12086-2)
Supplement: Supplementary file 5 — Supplementary file5 (DOCX 24 KB) [file 415_2023_12086_MOESM5_ESM.docx]

Table 1: Dystonia diagnosis inclusion criteria- ICD-10 and Read Codes V2. Table adapted from Wadon et al, 2022[1].

| **Dystonia subtype** | **ICD-10 Code** | **Read Code Description** | **Read code** |
| --- | --- | --- | --- |
| Idiopathic torsion dystonia |  | Idiopathic torsion dystonia | F136. |
|  |  | Symptomatic torsion dystonia | F137. |
|  |  | Symptomatic torsion dystonia OS | F137y |
|  |  | Symptomatic torsion dystonia NOS | F137z |
|  |  | Fragment of torsion dystonia | F138. |
|  |  | Torsion dystonia fragment NOS | F138z |
| Idiopathic nonfamilial dystonia | G24.2 |  |  |
| Idiopathic familial dystonia | G24.1 | Idiopathic familial dystonia | F1360 |
| Cervical dystonia | G24.3 | Spasmodic torticollis | F1382 |
|  |  | Torticollis - symptom | 16A3. |
|  |  | Torticollis unspecified | N135. |
|  |  | Intermittent torticollis | N1350 |
|  |  | Torticollis NOS | N135z |
| Idiopathic Orofacial dystonia | G24.4 |  |  |
| Blepharospasm | G24.5 | Blepharospasm | F1380 |
| Writer’s cramp |  | Organic Writer’s cramp | F1383 |
| Myoclonic dystonia |  | Myoclonic dystonia | F13B. |
| Segawa syndrome |  | Segawa syndrome | F13C. |
| Other | G24.8 | [X]Other dystonia | Fyu24 |
| Unspecified | G24.9 | [X]Dystonia, unspecified | Fyu2A |
|  |  | Dystonia, unspecified | F13X. |
| Tremor |  | Has a tremor | 1B22. |

*OS: otherwise specified, NOS not otherwise specified, EC: elsewhere classified, NEC: not elsewhere classified, FH: family history, O/E: on examination*

Table 2: Cohort diagnostic exclusion criteria for all participants using ICD-10 and Read code v2. Table adapted from Wadon et al, 2022 [1].

| **Diagnosis** | **Sub-diagnoses** | **Diagnostic codes** |
| --- | --- | --- |
| Dystonia | Drug induced dystonia | ICD-10: G24.0  Read codes: F1312 |
| Parkinsons disease and Parkinsonism | Parkinsons Disease; Parkinsons Disease NOS; O/E Parkinson gait; O/E Parkinson posture; O/E Parkinsonian tremor; Dementia in Parkinsons Disease; FH: Parkinsonism; Secondary Parkinsonism due to other external agents; Parkinsonism secondary to drugs; malignant neuroleptic syndrome; Post-encephalitic Parkinsonism; vascular Parkinsonism; Syphilitic Parkinsonism; secondary Parkinsonism Unspecified; secondary Parkinsonism; Other secondary Parkinsonism; Parkinsonism in Diseases EC; History of Parkinsons disease; Cerebral degeneration in Parkinsons Disease | ICD-10: G20; G21.0; G21.1; G21.2; G21.3; G21.4; G21.8; G21.9; G21; G22  Read codes: F12..; F12z.; 2994.; 2987.; 297A.; Eu023; 129Z.; F12W.; F121.; F122.; F123.; F124.; A94y1; F12X.; 147F.; F11x9 |
| Huntington’s Disease | Huntington’s chorea; Dementia in Huntington’s Disease; FH: Huntington’s chorea | ICD-10: G10  Read codes: F134.; Eu022; 1291. |
| Chorea | Other choreas; Hemiballismus; Paroxysmal chorea-athetosis; Drug-induced choreas; other choreas NOS | ICD-10: G25.5; G25.4  Read codes: F135.; F1350; F1351; F1352; F135z |
| Myoclonus | myoclonus | ICD-10: G25.3  Read codes: F132. |
| Ataxia | Cerebral ataxia; cerebellar ataxia NOS; cerebellar ataxia in diseases EC; cerebellar ataxia due to alcoholism; cerebellar ataxia due to myxoedema; cerebellar ataxia due to neoplasia; cerebellar ataxia in disease NOS; congenital non-progressive ataxia; Early onset cerebellar ataxia with hypogonadism; Friedreich’s ataxia; Spinocerebellar disease; spinocerebellar disease NOS; other spinocerebellar diseases; hereditary ataxia | ICD-10: G11  Read codes: F11y1; F143.; F144.; F1440; F1441; F1442; F144z; F145.; F146.; F140.; F14..; F14z.; F14y. |
| Degenerative diseases of the basal ganglia | Other basal ganglia degenerative diseases; Dejerine-Thomas syndrome; Hallervorden-Spatz Disease; Striatonigral degeneration; Parkinsonism with orthostatic hypotension; progressive supranuclear ophthalmoplegia; Shy-Drager syndrome; Acardi Goutieres syndrome; Other basal ganglia degenerative diseases NOS; Steele-Richardson-Olzewski syndrome; other specified degenerative diseases of basal ganglia; Degenerative disease of basal ganglia, unspecified | ICD-10: G23; G23.0; G23.1; G23.2; G23.8; G23.9  Read codes: F130.; F1300; F1301; F1302; F1303; F1304; F1305; F1306; F130z; F24y2 |
| Extrapyramidal diseases and movement disorders | Stiff man syndrome; restless leg syndrome; Akinetic rigid syndrome; hyperekplexia; Neuroferritinopathy; extrapyramidal disease and abnormal movement disorder NOS; other/unspecified extrapyramidal/abnormal movement disorders; Paroxysmal non-kinesigenic dyskinesia; paroxysmal kinesigenic dyskinesia | ICD-10:  Read codes: F13z1; F13z2; F13z3; F13z4; F13z6; F13zz; F139.; F1390; F1391 |
| Essential and other specified forms of tremor | Drug induced tremor; benign essential tremor | ICD-10: G25.1  Read codes: F1310; F1312 |
| Other cerebral degenerations | Alzheimer’s disease; Alzheimer’s disease with early onset; Alzheimer’s disease with late onset; Pick’s disease; Senile degeneration of brain; Lewy Body disease; Frontotemporal degeneration; Corticobasal degeneration | ICD-10:  Read codes: F110.; F1100; F1101; F111.; F112.; F116.; F118.; F11y2 |
| Hereditary and degenerative diseases of the central nervous system OS | Fragile X associated tremor ataxia syndrome; Hereditary and degenerative diseases of the central nervous system NOS | ICD-10:  Read codes: F1y0.; F1z.. |
| Demyelinating diseases of the central nervous system | Neimann-Pick disease; Progressive supranuclear palsy; Wilson’s disease; Multiple system atrophy; multiple system atrophy, cerebellar variant; multiple system atrophy, Parkinson variant | ICD-10: G23.3  Read codes: C3272; F24y0; C3510; F174.; F1740; F1741 |
| Tics/ tic disorders | Gilles de la Tourette’s disorder; combined vocal and multiple motor tic disorder [de la Tourette], Tic-symptom; O/E spasm/tic; Tic disorder unspecified; Transient childhood tic; Chronic motor tic disorder; Tic NOS; Tic disorders; transient tic disorder; chronic motor or vocal tic disorder; involuntary excessive blinking; other tic disorders; tic disorders, unspecified; tics; tics of organic origin | ICD-10: F95  Read codes: E2723; Eu952; 1B24.,;2974.; E2720; E2721; E2722; E272z; Eu95.; Eu950; Eu951; Eu953; Eu95y; Eu95z; E272.;F133. |
| Other degenerative diseases of the nervous system, NEC | Other degenerative diseases of the nervous system, NEC | ICD-10: G31 |
| Multiple sclerosis | Multiple sclerosis | ICD-10: G35 |
| Other acute disseminated demyelination | Other acute disseminated demyelination; other acute specified demyelination; neuromyelitis optica (devic) | ICD-10: G36; G36.0; G36.8; G36.9  Read codes: |
| Other demyelinating diseases of the central nervous system |  | ICD-10: G37 |
| Infantile cerebral palsy | Infantile cerebral palsy; spastic cerebral palsy; spastic diplegia; infantile hemiplegia; dyskinetic cerebral palsy; other infantile cerebral palsy; infantile cerebral palsy, unspecified | ICD-10: G80; G80.0; G80.1; G80.2; G80.3; G80.8; G80.9 |
|  |  |  |

*OS: otherwise specified, NOS not otherwise specified, EC: elsewhere classified, NEC: not elsewhere classified, FH: family history, O/E: on examination*

1. Wadon ME, Fenner E, Kendall KM, Bailey GA, Sandor C, Rees E, Peall KJ (2022) Clinical and genotypic analysis in determining dystonia non-motor phenotypic heterogeneity: a UK Biobank study. Journal of neurology
