# Supplementary material for: Macro- and micro-structural insights into primary dystonia: a UK Biobank study
Source: J Neurol. 2023 Nov 23;271(3):1416–27. doi: 10.1007/s00415-023-12086-2 (PMC10896800; doi:10.1007/s00415-023-12086-2)
Supplement: Supplementary file 6 — Supplementary file6 (DOCX 15 KB) [file 415_2023_12086_MOESM6_ESM.docx]

| ***ROI*** | ***Anatomical regions included*** |
| --- | --- |
| *Frontal cortex* | bilateral superior frontal gyri, rostral and caudal middle frontal gyri, pars opercularis, pars triangularis, pars orbitalis, lateral and medial orbitofrontal gyri, frontal pole and caudal and rostral anterior cingulate |
| *Sensorimotor cortex* | bilateral precentral gyri, postcentral gyri and paracentral lobule |
| *Non-frontal cortex* | bilateral superior and inferior parietal gyri, precuneus, supramarginal gyri, superior middle and inferior temporal gyri, fusiform gyri, transverse temporal gyri, enterorhinal gyri, temporal poles, parahippocampla gyri, lateral occipital gyri, cuneus, pericalcarine gyri and posterior cingulate gyri |
| *Striatum* | bilateral caudate, putamen and pallidum |
| *Thalami* | bilateral thalami |
| *Cerebellar cortex* | bilateral cerebellar cortices |

Supplementary Table 2: Grey matter regions of interest and the anatomical regions that they encompass. Abbreviations: ROI- region of interest.
